# MRI-Based Brain Tumor Classification Using Ensemble of Deep Features and Machine Learning Classifiers

**DOI:** 10.3390/s21062222

**Published:** 2021-03-22

**Authors:** Jaeyong Kang, Zahid Ullah, Jeonghwan Gwak

**Affiliations:** 1 Department of Software, Korea National University of Transportation, Chungju 27469, Korea; kjysmu@ut.ac.kr (J.K.); zahid@ut.ac.kr (Z.U.); 2 Department of Biomedical Engineering, Korea National University of Transportation, Chungju 27469, Korea; 3 Department of AI Robotics Engineering, Korea National University of Transportation, Chungju 27469, Korea; 4 Department of IT Convergence (Brain Korea PLUS 21), Korea National University of Transportation, Chungju 27469, Korea

**Keywords:** deep learning, ensemble learning, brain tumor classification, machine learning, transfer learning

## Abstract

Brain tumor classification plays an important role in clinical diagnosis and effective treatment. In this work, we propose a method for brain tumor classification using an ensemble of deep features and machine learning classifiers. In our proposed framework, we adopt the concept of transfer learning and uses several pre-trained deep convolutional neural networks to extract deep features from brain magnetic resonance (MR) images. The extracted deep features are then evaluated by several machine learning classifiers. The top three deep features which perform well on several machine learning classifiers are selected and concatenated as an ensemble of deep features which is then fed into several machine learning classifiers to predict the final output. To evaluate the different kinds of pre-trained models as a deep feature extractor, machine learning classifiers, and the effectiveness of an ensemble of deep feature for brain tumor classification, we use three different brain magnetic resonance imaging (MRI) datasets that are openly accessible from the web. Experimental results demonstrate that an ensemble of deep features can help improving performance significantly, and in most cases, support vector machine (SVM) with radial basis function (RBF) kernel outperforms other machine learning classifiers, especially for large datasets.

## 1. Introduction

In the human body, the brain is an enormous and complex organ that controls the whole nervous system, and it contains around 100-billion nerve cells [1]. This essential organ is originated in the center of the nervous system. Therefore, any kind of abnormality that exists in the brain may put human health in danger. Among such abnormalities, brain tumors are the most severe ones. Brain tumors are uncontrolled and unnatural growth of cells in the brain that can be classified into two groups such as primary tumors and secondary tumors. The primary tumors present in the brain tissue, while the secondary tumors expand from other parts of the human body to the brain tissue through the bloodstream [2]. Among the primary tumors, glioma and meningioma are two lethal types of brain tumors, and they may lead a patient to death if not diagnosed at an early stage [3]. In fact, the most common brain tumor in humans is glioma [4].

According to the World Health Organization (WHO), brain tumors can be classified into four grades [1]. The grade 1 and grade 2 tumors describe lower-level tumors (e.g., meningioma), while grade 3 and grade 4 tumors consist of more severe ones (e.g., glioma). In clinical practice, the incidence rates of meningioma, pituitary, and glioma tumors are approximately 15%, 15%, and 45%, respectively.

There are different ways to treat brain tumors depends on the tumor location, size, and type. Presently, the most common treatment for brain tumors is surgery as it has no side effects on the brain [5]. Different types of medical imaging technologies such as computed tomography (CT), positron emission tomography (PET), and magnetic resonance imaging (MRI) are available that are used to observe the internal parts of the human body conditions. Among all these imaging modalities, MRI is considered most preferable as it is the only non-invasive and non-ionizing modality that offers valuable information in 2D and 3D formats about brain tumor type, size, shape, and position [6]. However, manually reviewing these images is time-consuming, hectic, and even prone to error due to the influx of patients [7]. To address this problem, the development of an automatic computer-aided diagnosis (CAD) system is required to alleviate the workload of the classification and diagnosis of brain MRI and act as a tool for helping radiologists and doctors.

Several efforts have been made to develop a highly accurate and robust solution for the automatic classification of brain tumors. However, due to high inter and intra shape, texture, and contrast variations, it remains a challenging problem. The traditional machine learning (ML) techniques rely on handcrafted features, which restrains the robustness of the solution. Whereas the deep learning-based techniques automatically extract meaningful features which offer significantly better performance. However, deep learning-based techniques require a large amount of annotated data for training, and acquiring such data is a challenging task. To overcome these issues, in this study, we proposed a hybrid solution that exploits (1) various pre-trained deep convolutional neural networks (CNNs) as feature extractors to extract powerful and discriminative deep features from brain magnetic resonance (MR) images, and (2) various ML classifiers to identify the normal and abnormal brain MR images. Also, to investigate the benefits of combining features from different pre-trained CNN models, we designed the novel feature ensemble method for the MRI-based brain tumor classification task. We proposed the novel feature evaluation and selection mechanism where the deep features from 13 different pre-trained CNNs are evaluated using 9 different ML classifiers and selected based on our proposed feature selection criteria. In our proposed framework, we concatenated the selected top three deep features from three different CNNs to form a synthetic feature. The concatenation process integrates the information from different CNNs to create a more discriminative feature representation than using the feature extracted from a single CNN model since different CNN architectures can capture diverse information in brain MR images. An ensemble of deep features is then fed into several ML classifiers to predict the final output, whereas most of the previous works have employed traditional feature extraction techniques [8]. In our experiment, we provided an extensive evaluation using 13 different pre-trained deep convolutional neural networks and 9 different ML classifiers on three different datasets: (1) BT-small-2c, the small dataset with 2 classes (normal/tumor), (2) BT-large-2c, the large dataset with 2 classes (normal/tumor), and (3) the large dataset with 4 classes (normal, glioma tumor, meningioma tumor, and pituitary tumor) for brain tumor classification. Our experiment results demonstrate that the ensemble of deep features can help improving performance significantly. In summary, our contributions are listed as follows:

- We designed and implemented a fully automatic hybrid scheme for brain tumor classification, which uses both (1) the pre-trained CNN models to extract the deep features from brain MR images and (2) ML classifiers to classify brain tumor type effectively.
- We proposed a novel method which consists of three steps: (1) extract deep features using pre-trained CNN models for meaningful information extraction and better generalization, (2) select the top three performing features using fined-tuned several ML models for our task, and (2) combine them to build the ensemble model to achieve state-of-the-art performance for brain tumor classification in brain MR images.
- We conducted extensive experiments on 13 different pre-trained CNN models and 9 different ML classifiers to compare the effectiveness of each pre-trained CNN model and each ML classifier on three different brain MRI datasets: (1) BT-small-2c, the small dataset with 2 classes (normal/tumor), (2) BT-large-2c, the large dataset with 2 classes (normal/tumor), and (3) the large dataset with 4 classes (normal, glioma tumor, meningioma tumor, and pituitary tumor) for brain tumor classification.

The layout of this study is organized as follows: The related work is given in Section 2. The proposed method is presented in Section 3. The experimental settings and results are shown in Section 4. The conclusion section is described in Section 5.

## 2. Related Work

Numerous techniques have been proposed for automatic brain MRI classification based on traditional ML and deep learning methods as shown in Table 1.

The traditional ML methods are comprised of several steps: pre-processing, feature extraction, feature reduction, and classification. In traditional ML methods, feature extraction is a core step as the classification accuracy relies on extracted features. There are two main types of feature extraction. The first type of feature extraction is low-level (global) features, for instance, texture features and intensity, first-order statistics (e.g., mean, standard deviation, and skewness), and second-order statistics such as gray-level co-occurrence matrix (GLCM), wavelet transform (WT), Gabor feature, and shape. For instance, Selvaraj et al. [9] employed first-order and second-order statistics using least square support vector machine (SVM) and develop a binary classifier to classify the normal and abnormal brain MR images. John et al. [10] used GLCM and discrete wavelet transformation-based methods for tumor identification and classification. The low-level features represent the image efficiently; however, the low-level features and their representation capacity are limited since most brain tumors have similar appearances such as texture, boundary, shape, and size. Ullah et al. [8] extracted the approximation and detail coefficient of level-3 decomposition using DWT, reduced the coefficient by employing color moments (CM), and finally employed a feed-forward artificial neural network to identify the normal and abnormal brain MR images.

The second type of feature extraction is the high-level (local) features, such as fisher vector (FV), scale-invariant feature transformation (SIFT), and bag-of-words (BoW). Different researchers have employed BoW for medical image retrieval and classification. Such as the classification of breast tissue density in mammograms [11], X-ray images retrieval and classification on pathology and organ levels [12], and content-based retrieval of brain tumor [13]. Cheng et al. [14] employed FV to retrieve the brain tumor. The statistical features extracted from SIFT, FV, and BoW are high-level features formulated on a local scale that does not consider spatial information. Hence, it is noticeable that in the traditional ML method, there are two main problems in the feature extraction stage. First, it only focuses on either high-level or low-level features. Second, the traditional ML method depends on handcrafted features, which need strong prior information such as the location or position of the tumor in an image, and there are high chances of human errors. Therefore, it is essential to develop a method to combine both high-level and low-level features without using handcrafted features.

Most of the existing works in medical MR imaging refers to automatic segmentation of tumor region. Recently, Numerous researchers have proposed different techniques to detect and segment the tumor region in MR images [15,16,17]. Once the tumor in MRI is segmented, these tumors need to be classified into different grades. In previous research studies, binary classifiers have been employed to identify the benign and malignant classes [8,18,19]. For instance, Ullah et al. [8] proposed a hybrid scheme for the classification of brain MR images into normal and abnormal using histogram equalization, Discrete wavelet transform, and feed-forward artificial neural network, respectively. Kharrat et al. [18] categorize the brain tumor into normal and abnormal using a genetic algorithm and support vector machine. Besides, Papageorgiou et al. [19] categorized the high-grade and low-grade gliomas based on fuzzy cognitive maps and attained 93.22% and 90.26% accuracy for high-grade and low-grade brain tumors, respectively.

Shree and Kumar [20] divided the brain MRI into two classes: normal and abnormal. They used GLCM for feature extraction, while a probabilistic neural network (PNN) classifier has been employed to classify the brain MR image into normal and abnormal and obtained 95% accuracy. Arunachalam and Savarimuthu [21] proposed a model to categorize the normal and abnormal brain tumor in brain MR images. Their proposed model comprised enhancement, transformation, feature extraction, and classification. First, they have enhanced the brain MR image using shift-invariant shearlet transform (SIST). Then, they extracted the features using Gabor, grey level co-occurrence matrix (GLCM), and discrete wavelet transform (DWT). Finally, these extracted features were then fed into feed-forward backpropagation neural network and obtained a high accuracy rate. Rajan and Sundar [22] proposed a hybrid energy-efficient method for automatic tumor detection and segmentation. Their proposed method is comprised of seven long phases and reported 98% accuracy. The main drawback of their proposed model is high computation time due to the use of numerous techniques.

Since the last decade, deep learning methods have been widely used for brain MRI classification [23,24]. The deep learning method does not need handcrafted (manually) extracted features as it embedded the feature extraction and classification stage in self-learning. The deep learning method requires a dataset where sometimes a pre-processing operation needs to be done, and then salient features are determined in a self-learning manner [25]. In MR imaging classification, a key challenge is to reduce the semantic gap between the high-level visual information perceived by the human evaluator and the low-level visual information captured by the MR imaging machine. To reduce the semantic gap, the convolutional neural networks (CNNs), one of the famous deep learning techniques for image data, can be used as a feature extractor to capture the relevant features for the classification task. Feature maps in the initial layers and higher layers of CNNs models extract low-level features and high-level content (domain) specific features, respectively. Feature maps in the earlier layer construct simple structural information, for instance, shape, textures, and edges, whereas higher layers combine these low-level features to construct (encode) efficient representation, which integrates global and local information.

Recently, different researchers have used CNNs for brain MRI classification and validated their proposed methodology on brain tumor classification datasets [26,27,28]. Deepak and Ameer [29] used a pre-trained GoogLeNet to extract features from brain MR images with deep CNN to classify three types of brain tumor and obtained 98% accuracy. Ahmet and Muhammad [30] used different CNN models such as GoogLeNet, Inception V3, DenseNet-201, AlexNet, and ResNet-50 to classify the brain MR images and obtained reasonable accuracies. They modified pre-trained ResNet-50 CNN by removing its last 5 layers and added new 8 layers, and obtained 97.2% accuracy with this model, which is the highest accuracy among all pre-trained models. Khwaldeh et al. [31] proposed a CNN model to classify the normality and abnormality of brain MR images as well as high-grade and low-grade glioma tumors. They have modified the AlexNet CNN model and used it as their network architecture, and they obtained 91% accuracy. Despite the valuable works being done in this area, developing a robust and practical method still requires more effort to classify brain MR images. Saxena et al. [32] used Inception V3, ResNet-50, and VGG-16 models with transfer learning methods to classify brain tumor data. The ResNet-50 model obtained the highest accuracy rate with 95%. In studies [33,34] CNN architectures have been introduced to classify brain tumors. In these architectures, the convolution neural network extracts the features from brain MRI using convolution and pooling operations. The main purpose of these proposed models is to find the best deep learning model that accurately classifies the brain MR images. Francisco et al. [35] presented a multi-pathway CNN architecture for automatic brain tumor segmentation such as glioma, meningioma, and pituitary tumor. They have evaluated their proposed model using a publicly available T1-weighted contrast-enhanced MRI dataset and obtained 97.3% accuracy. However, their training procedure is quite expensive. Raja et al., [36] proposed a hybrid deep autoencoder (DAE) for brain tumor classification using the Bayesian fuzzy clustering (BFC) approach. Initially, they have used a non-local mean filter to remove the noise from the image. Then the BFC approach is employed for brain tumor segmentation. Furthermore, some robust features were extracted using scattering transform (ST), information-theoretic measures, and wavelet packet Tsallis entropy (WPTE). Eventually, a hybrid scheme of DAE is employed for brain tumor classification and achieved high accuracy. The main drawback of this approach is, it requires high computation time due to the complex proposed model.

In summary, as observed from the above research studies, the acquired accuracies using deep learning techniques for brain MRI classification are significantly high as compared to traditional ML techniques. However, the deep learning models require a massive amount of data for training in order to perform better than traditional ML techniques.

It is clearly seen from recently published studies that deep learning techniques have become one of the mainstream of expert and intelligent systems and medical image analysis. Furthermore, the techniques mentioned earlier have certain limitations which should be considered while working with brain tumor classification and segmentation. The major drawback of the previously proposed systems is that they only consider binary classification (normal and abnormal) MR image dataset and ignore the multi-class dataset [37]. In the pre-screening stage of a patient, binary class classification is required for physicians and radiologists, where the physicians take further action based on binary class classification. Preethi and Aishwarya [38] proposed a model to classify the brain tumor based on multiple stages. They combined the wavelet-based gray-level co-occurrence matrix and GLCM to produce the feature matrix. The extracted features were further reduced using the oppositional flower pollination algorithm (OFPA). Finally, the deep neural network is employed to classify the MR brain image based on the selected features and obtained 92% accuracy. Ural [39] initially enhanced the brain MRI using different image processing techniques. Also, different segmentation process has been mixed for boosting the performance of the solution. Further, the PNN method is employed to detect and localize the tumor area in the brain. The computational time of their proposed method is quite low and also the acquired accuracy rate is quite reasonable.

## 3. Proposed Methods

In this section, the overall architecture of our proposed method is first described. After that, we describe the details of four key components in the following subsections.

**Figure 1:**
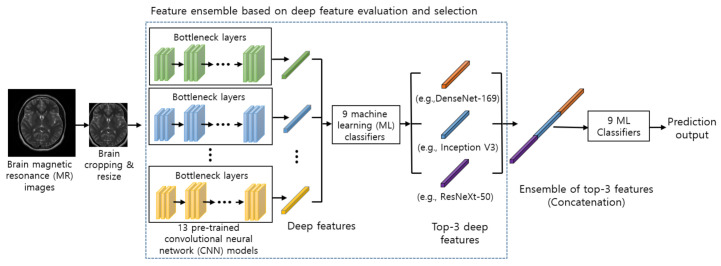
Architecture of our proposed model using feature ensemble based on deep feature evaluation and selection.

The architecture of our proposed method for brain tumor classification is illustrated in Figure 1. First, input MR images are pre-processed (e.g., brain cropping, resize, and augmentation) before feeding into the model (Section 3.1). Second, the pre-processed images are used as the input of pre-trained CNN models as feature extractors (Section 3.2). The extracted features from pre-trained CNN models are evaluated by several ML classifiers. (Section 3.3). The top three deep features are selected based on evaluation results from the classifiers (Section 3.4). The top three deep features are concatenated in our ensemble module, and the concatenated deep features are further used as an input to ML classifiers to predict final output (Section 3.5).

### 3.1. Image Pre-Processing

**Figure 2:**
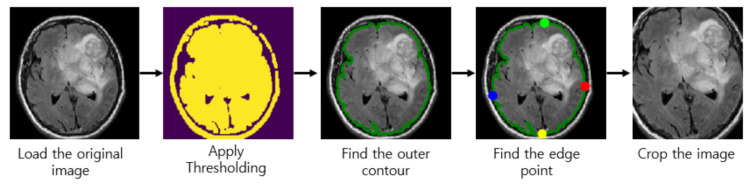
Step to crop the magnetic resonance (MR) images.

Almost every image in our brain MRI datasets contains undesired spaces and areas, leading to poor classification performance. Hence, it is necessary to crop the images to remove unwanted areas and use only useful information from the image. We use the cropping method in [40] which uses extreme point calculation. The step to crop the MR images using extreme point calculation is shown in Figure 2. First, we load the original MR images for pre-processing. After that, we apply thresholding to the MR images to convert them into binary images. Also, we perform the dilation and erosions operations to remove the noise of images. After that, we selected the largest contour of the threshold images and calculated the four extreme points (extreme top, extreme bottom, extreme right, and extreme left) of the images. Lastly, we crop the image using the information of contour and extreme points. The cropped tumor images are resized by bicubic interpolation. The specific reason to choose the bicubic interpolation is that it can create a smoother curve than other interpolation methods such as bilinear interpolation and is a better choice for MR images since there is a large amount of noise along the edges.

Also, we used image augmentation since the size of our MRI dataset is not very large. Image augmentation is the technique that creates an artificial dataset by modifying the original dataset. It is known as the process of creating multiple copies of the original image with different scales, orientation, location, brightness, and so on. It is reported that the classification accuracy of the model can be improved by augmenting the existing data rather than collecting new data.

In our image augmentation step, we used 2 augmentation strategies (rotation and horizontal flipping) to generate new training sets. The rotation operation used for data augmentation is done by randomly rotating the input by 90 degrees zero or more times. Also, we applied horizontal flipping to each of the rotated images.

Since the MR images in our dataset are of different width, height, and sizes, it is recommended to resize them to equal width and height to get optimum results. In this work, we resize the MR images to the size of either 224 × 224 (or 299 × 299) pixels since input image dimensions of pre-trained CNN models are 224 × 224 pixels except for the Inception V3, which requires the input images with size 299 × 299.

### 3.2. Deep Feature Extraction Using Pre-Trained CNN Models

#### 3.2.1. Convolutional Neural Network

CNN is a class of deep neural networks that uses the convolutional layers for filtering inputs for useful information. The convolutional layers of CNN apply the convolutional filters to the input for computing the output of neurons that are connected to local regions in the input. It helps in extracting the spatial and temporal features in an image. A weight-sharing method is used in the convolutional layers of CNN to reduce the total number of parameters [41,42].

**Figure 3:**
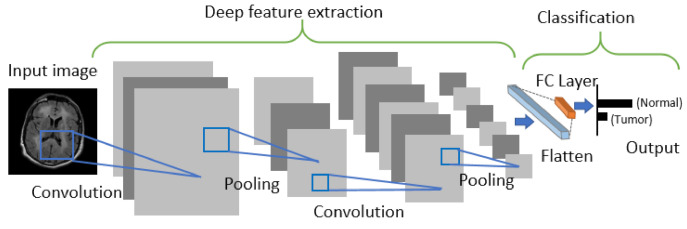
Architecture of Convolutional Neural Networks.

CNN is generally comprised of three building blocks: (1) a convolutional layer to learn the spatial and temporal features, (2) a subsampling (max-pooling) layer to reduce or downsample the dimensionality of an input image, and (3) a fully connected (FC) layer for classifying the input image into various classes. The architecture of CNN is shown in Figure 3.

#### 3.2.2. Transfer Learning

**Figure 4:**
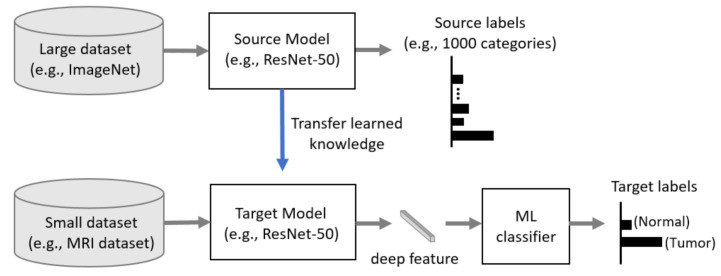
Concept of transfer learning.

Generally, CNN has better performance in a larger dataset than a smaller one. Transfer learning can be used when it is not feasible to create a large training dataset. The concept of transfer learning can be depicted in Figure 4, where the model pre-trained on large benchmark datasets (e.g., ImageNet [43]) can be used as a feature extractor for the different task with a relatively smaller dataset such as an MRI dataset. In recent years, transfer learning technique has been successfully applied in various domains, such as medical image classification and segmentation, and X-ray baggage security screening [44,45,46,47]. This reduces the long training time that is normally required for training deep learning models from scratch and also removes the requirement of having a large dataset for the training model [48,49].

#### 3.2.3. Deep Feature Extraction

In this study, we use a CNN-based model as a deep learning-based feature extractor since it can capture the important features without any human supervision. Also, we use a transfer learning-based approach to build our feature extractor since our MRI dataset is not very large and training and optimizing deep CNN such as DenseNet-121 from scratch is often not feasible. Hence, we use the fixed weights of each CNN model pre-trained on a large ImageNet dataset to extract the deep features of brain MR images.

The pre-trained CNN models used in our study are ResNet [50], DenseNet [51], VGG [52], AlexNet [53], Inception V3 [54], ResNeXt [55], ShuffleNet V2 [56], MobileNet V2 [57], and MnasNet [58]. The extracted deep features are then fed into the ML classifiers, including neural networks with a FC layer as a traditional deep learning approach using CNN as shown in Figure 3 to predict the output.

### 3.3. Machine Learning Classifiers for Brain Tumor Classification

The extracted deep features from pre-trained CNN models are used as an input of several ML classifiers, including neural networks with an FC layer, Gaussian Naïve Bayes (Gaussian NB), Adaptive Boosting (AdaBoost), K-Nearest Neighbors (k-NN), Random forest (RF), SVM with three different kernels: linear, sigmoid, and radial basis function (RBF), Extreme Learning Machine (ELM). We implemented these ML classifiers using the scikit-learn ML library [59]. These ML classifiers and their hyper-parameter settings used in our experiments for brain tumor classification are discussed in the following subsections.

#### 3.3.1. Fully Connected Layer

In neural networks with an FC layer, which is the traditional deep learning approach, the loss function is defined to calculate the loss, which is a prediction error of the neural network. The loss is used to calculate the gradients to update the weights of the neural network as a training step. In our training step of the FC classifier, we use the cross-entropy loss function, which is the most commonly used loss function for CNN and other neural networks. It calculates the loss between the soft target estimated by the softmax function and the ground-truth label to learn our model parameters as follows:

(1) $$L\left(y,z\right)=\sum_{i=0}^{M}-y_{i}\log\left(\frac{z_{i}}{\sum_{j}exp\left(z_{i}\right)}\right)$$

where *M* is the total number of class, for instance, M is set to 2 when the classifier is trained on the two MRI datasets, BT-small-2c and BT-large-2c, which contain two classes (normal and tumor) of MR images, and M is set to 4 when the classifier is trained on the MRI dataset, BT-large-4c, which contains four classes (normal, glioma tumor, meningioma tumor, and pituitary tumor) of MR images (See Section 4.1 for the details of these datasets), *y* is a one-hot encoded vector representing the ground-truth label of the training set as 1 and all other elements as 0, and $z_{i}$ is the logit which is the output of the last layer for the *i*-th class of the model.

In this work, we update the weight of the layers via Adaptive Moment Estimation (Adam), the optimizer that calculates the adaptive learning rates of every parameter. The learning rate is set to 0.001. We run each of the methods for 100 epochs. We collect the highest average accuracy for our test dataset for each run.

#### 3.3.2. Gaussian Naïve Bayes

Naïve Bayes classifier is the ML classifier with the assumption of conditional independence between the attributes given the class. In this work, we use Gaussian NB classifier as one of our ML classifiers for brain tumor classification. In Gaussian NB classifier, the conditional probability *P(y|X)* is calculated as a product of the individual conditional probabilities using the naïve independence assumption as follows:

(2) $$P\left(y|X\right)=\frac{P(y)P(X|y)}{P(X)}=\frac{P\left(y\right)\prod_{i=1}^{n}P\left(x_{i}|y\right)}{P(X)}$$

where *X* is given data instance (extracted deep feature from brain MR image) which is represented by its feature vector $\left(x_{1},\ldots ,x_{n}\right)$, *y* is a class target (type of brain tumor) with two classes (normal and tumor) for two MRI datasets, BT-small-2c and BT-large-2c, or four classes (normal, glioma tumor, meningioma tumor, and pituitary tumor) for BT-large-4c dataset. Since P(X) is constant, the given data instance can be classified as follows:

(3) $$\hat{y}=\underset{y}{\mathrm{error}}P\left(y\right)\prod_{i=1}^{n}P\left(x_{i}|y\right)$$

where $\left(x_{i}|y\right)$ is calculated assuming that the likelihood of features to be Gaussian as follows:

(4) $$P\left(x_{i}|y\right)=\frac{1}{\sqrt{2\pi \sigma_{y}^{2}}}exp\left(\frac{{\left(x_{i}-\mu_{y}\right)}^{2}}{2\sigma_{y}^{2}}\right)$$

where the parameters $\mu_{y}$ and $\sigma_{y}$ are estimated using maximum likelihood.

In this work, the smoothing variable representing the portion of the largest variance of all features that are added to variances for calculation stability is set to $10^{-9}$, the default value of the scikit-learn ML library.

#### 3.3.3. AdaBoost

AdaBoost, proposed by Freund and Schapire [60], is an ensemble learning algorithm that combines multiple classifiers to improve performance. AdaBoost classifier builds a well-performing strong classifier by combining multiple weak classifiers using the iterative ensemble method. The underlying idea of Adaboost is to set the weights of classifiers and train the data sample in each boosting iteration to accurately predict a class target (a type of brain tumor) of a given data instance (extracted deep feature from brain MR image) with two classes (normal and tumor) for two MRI datasets, BT-small-2c and BT-large-2c, or four classes (normal, glioma tumor, meningioma tumor, and pituitary tumor) for BT-large-4c dataset. Any ML classifier that accepts the weights on the training set can be used as a base classifier.

In this work, we adopt the decision tree classifier as our base classifier since it is a commonly used base classifier for AdaBoost. Also, the number of the estimator is set to 150.

#### 3.3.4. K-Nearest Neighbors

k-NN is one of the simplest classification techniques. It performs predictions directly from the training set that is stored in the memory. For instance, to classify a new data instance (a deep feature from brain MR image), k-NN chooses the set of *k* objects from the training instances that are closest to the new data instance by calculating the distance and assigns the label with two classes (normal or tumor) or four classes (normal, glioma, meningioma, and pituitary tumor) and does the selection based on the majority vote of its *k* neighbors to the new data instance.

Manhattan distance and Euclidean distance are the most commonly used to measure the closeness of the new data instance with the training data instances. In this work, we used the Euclidean distance measure for the k-NN algorithm. Euclidean distance *d* between data point *x* and data point *y* are calculated as follows:

(5) $$d\left(x,y\right)=\sqrt{\left(\sum_{i=1}^{N}{\left(x_{i}-y_{i}\right)}^{2}\right)}$$

The brief summary of k-NN algorithm is illustrated below:

- First select a suitable distance metric.
- Store all the training data set *P* in pairs in the training phase as follows:
  (6) $$P=(y_{i},c_{i}),i=1,\ldots ,n$$
  where in the training dataset, $y_{i}$ is a training pattern, *n* is the amount of training patterns and $c_{i}$ is its corresponding class.
- In the testing phase, compute the distances between the new features vector and the stored (training data) features, and classify the new class example by a majority vote of its k neighbors.

The correct classification given in the test phase is used to evaluate the accuracy of the algorithm. If the result is not satisfactory, the *k* value can be adjusted until a reasonable level of accuracy is obtained. It is noticeable here that we set the number of neighbors from 1 to 4 and selected the one with the highest accuracy.

#### 3.3.5. Random Forest

RF, proposed by Breiman [61], is an ensemble learning algorithm that builds multiple decision trees using the bagging method to classify new data instance (a deep feature of brain MR image) to a class target (a type of brain tumor) with two classes (normal and tumor) for two MRI datasets, BT-small-2c and BT-large-2c, or four classes (normal, glioma tumor, meningioma tumor, and pituitary tumor) for BT-large-4c dataset. RF selects random *n* attributes or features to find the optimal split point using the Gini index as a cost function while creating the decision trees. This random selection of the attributes or features can reduce the correlation among the trees and have lower ensemble error rates. The new observation is fed into all classification trees of the RF for predicting a class target (a type of brain tumor) of the new incoming data instance. RF counts the numbers of predictions for each class and selects the class with the largest number of votes as the class label for the new data instance.

In this work, the number of features to consider when looking for the best split is set to the square root of the total number of features. Also, we set the number of trees from 1 to 150 and selected the one with the highest accuracy.

#### 3.3.6. Support Vector Machine

SVM, proposed by Vapnik [62], is one of the most powerful classification algorithms. SVM uses the kernel function, $K(x_{n},x_{i})$, to transform the original data space into an another space with a higher dimension. The hyperplane function for separating the data can be defined as follows:

(7) $$f\left(x_{i}\right)=\sum_{n=1}^{N}\alpha_{n}y_{n}K\left(x_{n},x_{i}\right)+b$$

where $x_{n}$ is support vector data (deep features from brain MR image), $\alpha_{n}$ is Lagrange multiplier, and $y_{n}$ represent a target class of these three datasets employed in this paper, such that the two datasets are binary (normal and abnormal) class datasets, while the third dataset has four different classes (normal, glioma, meningioma, and pituitary tumor) with n=1,2,3,…,N.

In this work, we used the most commonly used kernel functions at the SVM algorithm: (1) linear kernel, (2) sigmoid kernel, and (3) RBF kernel. Table 2 shows the details of three kernels. Also, SVM has two key hyper-parameters, C and Gamma. C is the hyper-parameter for the soft margin cost function that controls the influence of each support vector. Gamma is the hyper-parameter that decides how much curvature we want in a decision boundary. We set the gamma and C values to [0.00001, 0.0001, 0.001, 0.01] and [0.1, 1, 10, 100, 1000, 10000], respectively, and selected the combination of gamma and C values with the highest accuracy.

#### 3.3.7. Extreme Learning Machine (ELM)

Extreme Learning Machine (ELM) is a simple learning algorithm for single-hidden layer feed-forward neural networks (SLFNs). ELM was initially proposed by Huang et al. [63] to overcome the limitations of traditional SLFNs learning algorithms, such as poor generalization effectiveness, irrelevant parameter tuning, and slow learning speed. ELM has shown a considerable ability for regression and classification tasks with good generalization performance.

In ELM, the output of a SLFN with $\tilde{N}$ hidden nodes given *N* distinct training samples, can be represented as follows:

(8) $$o_{j}=\sum_{i=1}^{\tilde{N}}\beta_{i}f_{i}\left(x_{j}\right)=\sum_{i=1}^{\tilde{N}}\beta_{i}f\left(x_{j};a_{i},b_{i}\right),j=1,\ldots ,N$$

where $o_{j}$ is the output vector of the SLFN, which represents the probability of the input sample $x_{i}$ (deep features from brain MR image) belonging to a class target (type of brain tumor) with two classes (normal and tumor) for two MRI datasets, BT-small-2c and BT-large-2c, or four classes (normal, glioma tumor, meningioma tumor, and pituitary tumor) for BT-large-4c dataset, $a_{i}$ and $b_{i}$ are learning parameters generated randomly of the *j*-th hidden node, respectively, $\beta_{i}$ is the link connecting the *j*-th hidden node and the output nodes, and $f(x_{j};a_{i},b_{i})$ is the activation function of ELM.

The ELM learning algorithm can be explained in 3 steps. First, the parameters (weights and biases) of all neurons are randomly initialized. Second, the hidden layer output matrix of the neural network *H* is calculated. Third, the output weight, β is calculated as follows:

(9) $$\beta =H^{\prime }T$$

where $H^{\prime }$ is the Moore-Penrose generalized inverse of matrix H (the hidden layer output matrix), which can be obtained by minimum-norm least-squares solution, and *T* is the target matrix corresponding to *H*.

In this work, the number of the hidden layer is set to [5000, 6000, 7000, 8000, 9000, 10,000], and select the one with the highest accuracy.

#### 3.3.8. Discussion

Several efforts have been made to develop a highly accurate and robust solution for MRI-based brain tumor classification using various ML classifiers: neural network classifier [8,21,64], Naïve Bayes classifier [65], AdaBoost classifier [66], k-NN classifier [64], RF classifier [64,67], SVM classifier [18,22], and ELM classifier [68]. However, there have been no studies done on evaluating the effectiveness of ML classifiers for the MRI-based brain tumor classification task. Hence, in our study, we use 9 well-known different ML classifiers to examine which ML classifier performs well for the MRI-based brain tumor classification task.

Since the performance of ML classifiers are highly dependent on input feature map, designing a method to produce a discriminative and informative feature from brain MR images plays a key role to successfully build the model for MRI-based brain tumor classification. In recent years, several studies proposed deep-learning-based feature extraction methods for MRI-based brain tumor classification using pre-trained deep CNN models: ResNet-50 [69,70], ResNet-101 [71], DenseNet-121 [70,72], VGG-16 [69,70], VGG-19 [70,73], AlexNet [74], Inception V1 (GoogLeNet) [29], Inception V3 [69,75], and MobileNet V2 [76]. However, no study has been carried out to evaluate the effectiveness of several pre-trained deep CNN models as a feature extractor for MRI-based brain tumor classification task. Hence, we use 13 different pre-trained deep CNN models to examine which pre-trained CNN models are useful as a feature extractor for MRI-based brain tumor classification task.

### 3.4. Deep Feature Evaluation and Selection

We evaluate each deep feature extracted from 13 different pre-trained CNNs using 9 different ML classifiers (FC, Gaussian NB, AdaBoost, k-NN, RF, SVM-linear, SVM-sigmoid, SVM-RBF, and ELM) described in Section 3.3 and choose the top three deep features based on the average accuracy of 9 different ML classifiers for each of our 3 different MRI datasets. In case the accuracy of two or more deep features is the same, we choose the one with the lowest standard deviation. Also, if there are more than 2 deep features extracted from two homogeneous pre-trained models (e.g., DenseNet-121 and DenseNet-169) among the top three features, we exclude the one with lower accuracy and choose the next best deep feature. The reason for doing this is that the deep features extracted from two homogeneous models share similar feature spaces. Hence, the ensemble of these features has redundant feature space and a lack of diversity. The top three deep features are fed into our ensemble module described in the following sub-section.

### 3.5. Ensemble of Deep Features

Ensemble learning aims at improving the performance and prevents the risk of using a single feature extracted from one model with a poor performance by combining multiple features from several different models into one predictive feature. Ensemble learning can be divided into feature ensemble and classifier ensemble depending on integration level. Feature ensemble involves integrating feature sets that are further fed to the classifier for final output, while classifier ensemble involves integrating output sets from classifiers where voting methods determine the final output. Since the feature set contains richer information about the MR images than the output set of each classifier, integration at this level is expected to provide better classification results. Hence, in this work, we use feature ensemble as our ensemble learning.

In our ensemble module, we concatenate the top three deep features from three different pre-trained CNNs as one sequence. For instance, in Figure 1, the top three deep features are DenseNet-169, Inception V3, and ResNeXt-50, and these features are concatenated into one sequence as our feature-level ensemble step. The concatenated deep feature is further fed to ML classifiers for predicting the final output. Also, we concatenate all the possible combinations of two features from the top three features, which is further fed to ML classifiers to compare with the model using the ensemble of the top three features in our experiments.

## 4. Experiments and Results

### 4.1. Dataset

**Figure 5:**
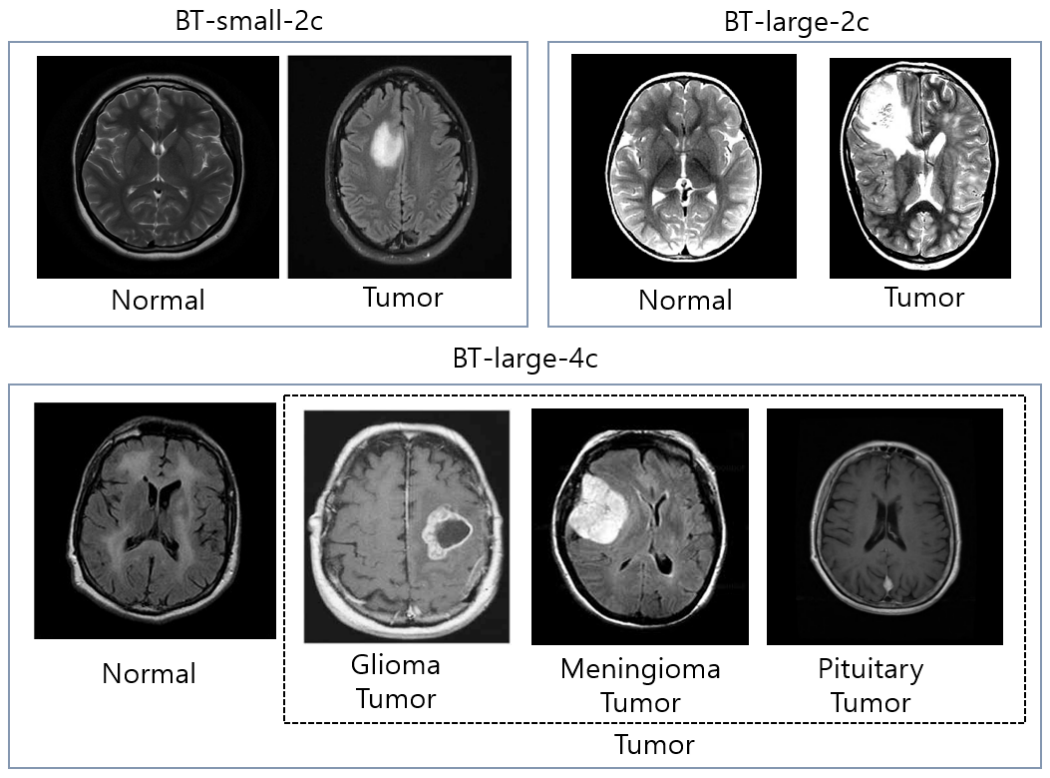
The examples of brain MR images in BT-small-2c, BT-large-2c, and BT-large-4c datasets.

We perform a set of experiments on three different brain MRI datasets which are publicly available for the tasks of brain tumor classification. The first dataset of brain MR images was downloaded from the Kaggle website [77], and for our simplicity, we named this dataset BT-small-2c. The BT-small-2c dataset comprises 253 images, out of which 155 images contain tumors while the remaining 98 images are without tumors. The second dataset was also downloaded from the Kaggle website, namely Brain Tumor Detection 2020 [78], and we call it BT-large-2c. This database comprises 3000 images, out of which 1500 images contain tumors while the remaining 1500 images are without tumors. The third dataset consists of 3064 T1-weighted images containing three different types of brain tumors such as gliomas, meningiomas, and pituitary tumors. The dataset was acquired from the Kaggle website [37], and we named this dataset as BT-large-4c. The BT-small-2c and BT-large-2c datasets contain brain MR images with two classes (normal and tumor). The BT-large-4c dataset contains brain MR images with four classes (normal, glioma tumor, meningioma tumor, and pituitary tumor). Each dataset is subdivided into a training set (80% of the total dataset) and a test set (20% of the total dataset). Table 3 shows details of the dataset used in our experiments. The examples of brain MR images in BT-small-2c, BT-large-2c, and BT-large-4c datasets are shown in Figure 5.

### 4.2. Experimental Setting

In our experiment, we use 13 different pre-trained deep convolutional neural networks as a feature extractor: ResNet-50, ResNet-101, DenseNet-121, DenseNet-169, VGG-16, VGG-19, AlexNet, Inception V3, ResNext-50, ResNext-101, ShuffleNet, MobileNet, MnasNet. We freeze the weight of bottleneck layers of deep CNN models pre-trained on the ImageNet [79] dataset. Also, we use 9 different ML classifiers: FC layer, Gaussian NB, AdaBoost, k-NN, RF, SVM with three different kernels (linear, sigmoid, and RBF), ELM. Before the training step, we pre-processed the input images as described in Section 3.1. Also, we converted the images to the size 224 × 224 (or 299 × 299) pixels as the pre-trained networks used in our experiments require the input images with size 224 × 224 except for the Inception V3, which requires the input images with size 299 × 299. All experiments were performed on a PC with an NVIDIA GeForce GTX 1070 Ti GPU.

### 4.3. Results

The empirical results were obtained for three different datasets (BT-small-2c, BT-large-2c, and BT-large-4c) for the tasks of the brain tumor classification. The first experiment is designed to compare the several different pre-trained CNN networks with several different ML classifiers. The second experiment is designed to show the effectiveness of the ensemble of top 2 or 3 deep features selected by the results from the first experiment with several different ML classifiers. The results of the first experiments on BT-small-2c, BT-large-2c, and BT-large-4c datasets are shown in Table 4, Table 5 and Table 6, respectively. As shown in Table 4, DenseNet-169 feature, Inception V3 feature, and ResNeXt-50 feature are selected as the top three deep features on BT-small-2c dataset. As shown in Table 5, DenseNet-121 feature, ResNeXt-101 feature, and MnasNet feature are selected as the top three deep features on BT-small-4c dataset. Also in Table 6, DenseNet-169 feature, ShuffleNet V2 feature, and MnasNet feature are selected as the top three deep features on BT-large-4c dataset.

Also, the results of the second experiments on BT-small-2c, BT-large-2c, and BT-large-4c datasets are shown in Table 7, Table 8 and Table 9, respectively. Also, the computational complexity of ensemble models is compared based on the inference time on a test set of the BT-large-4c dataset as shown in Table 10. From these results, five observations were made.

– *Observation 1*. SVM with RBF kernel outperforms other ML classifiers on two large datasets (BT-large-2c and BT-large-4c).
– *Analysis*. Table 5 and Table 6 show that the SVM with RBF kernel outperforms other ML classifiers on two large datasets (BT-large-2c and BT-large-4c). This is because SVM with RBF kernel can find a more effective and complex set of decision boundaries than other ML classifiers. However, as you can see in Table 4, SVM with RBF kernel does not outperform other ML classifiers on the small dataset. This is because SVM tends to underperform when the number of features for each data point is larger than the number of training data samples.
– *Observation 2*. Gaussian NB performs worst among other ML classifiers on three datasets.
– *Analysis*. Table 4, Table 5 and Table 6 show that Gaussian NB performs worst among other ML classifiers on three datasets. This is because Gaussian NB assumes the features are independent. However, it is almost impossible that the extracted features from the pre-trained models are completely independent.
– *Observation 3*. The deep feature from DenseNet architectures performs well than the deep features from other pre-trained CNN networks, while the deep features from VGG perform worse than the deep features from other pre-trained CNN networks on three different datasets.
– *Analysis*. Table 4, Table 5 and Table 6 show that the deep feature from DenseNet architectures performs well than the deep features from other pre-trained CNN networks on three different datasets. This is because the features extracted from DenseNet have all complexity levels. Hence, it tends to give more smooth decision boundaries, which can predict well when training data is insufficient. On the other hand, the deep feature from VGG performs worse than the deep features from other pre-trained CNN networks on three different datasets. This is because VGG is a more basic architecture that uses no residual blocks than other pre-trained CNN networks.
– *Observation 4*. Using the ensemble of deep features from two or three pre-trained CNN models is effective for all ML classifiers on a large dataset. However, the ensemble of deep features is effective for only ML classifiers on a small dataset.
– *Analysis*. Table 8 and Table 9 show that the model with the ensemble of deep features from two or three pre-trained CNN models achieves higher accuracy than the model with a deep feature from an individual pre-trained CNN model. This is because the ensemble model takes advantages of well-performing top-2 or 3 deep features by concatenating them, and also the concatenation of these deep features has a set of features that are capable of representing the data present in the images in a different way which benefits to improve the performance of ML classifiers. However, Table 7 shows that the ensemble of deep features is effective for only a few ML classifiers on a small dataset. This is because the number of the training sample is not enough in the small dataset to learn the complex set of the ensemble of deep features.
– *Observation 5*. k-NN classifier takes the longest time for inference on a test set while FC, Gaussian NB, and RF take a shorter inference time.
– *Analysis*. Table 10 shows that the k-NN classifier takes the longest time for inference on a test set among other ML classifiers while FC, Gaussian NB, and RF classifiers take a very short time for inference on a test set. This is because the k-NN classifier has to look at all the data points to make a single prediction, whereas other ML classifiers are not dependent on the number of training data points on the predict phase. On the other hand, the Gaussian NB classifier uses the Bayes equation to compute the posterior probabilities for inference. This involves trivial arithmetic operations such as multiplication and addition. Also, normalization is done by simple division operations. In the fully connected layer (FC), the entire matrix calculation for inference can be done by fast GPU. RF classifier leverages the power of multiple decision trees, which are simple and fast for making decisions. Therefore, these three classifiers achieved significantly less computation time for inference than other ML classifiers.

## 5. Conclusions

In summary, we presented a brain tumor classification method using the ensemble of deep features from pre-trained deep convolutional neural networks with ML classifiers. In our proposed framework, we use several pre-trained deep convolutional neural networks to extract deep features from brain MR images. The extracted deep features are then evaluated by several ML classifiers. The top three deep features which perform well on several ML classifiers are selected and concatenated as an ensemble of deep feature which is then fed into several ML classifiers to predict the final output. In our experiment, we provided an extensive evaluation using 13 different pre-trained deep convolutional neural networks and nine different ML classifiers on three different datasets (BT-small-2c, BT-large-2c, and BT-large-4c) for brain tumor classification. Our experiment results indicate that from our architecture, (1) DenseNet-169 deep feature alone is a good choice in case the size of the MRI dataset is very small and the number of classes is 2 (normal, tumor), (2) the ensemble of DenseNet-169, Inception V3, and ResNeXt-50 deep features is a good choice in case the size of MRI dataset is large and the number of classes is 2 (normal, tumor) and (3) the ensemble of DenseNet-169, ShuffleNet V2, and MnasNet deep features is a good choice in case the size of MRI dataset is large and there are four classes (normal, glioma tumor, meningioma tumor, and pituitary tumor). Also, in most cases, SVM with RBF kernel outperforms other ML classifiers for the MRI-based brain tumor classification task. In summary, our proposed novel feature ensemble method helps to overcome the limitations of a single CNN model and produces superior and robust performance, especially for large datasets. These results indicated that our proposed method using an ensemble of deep features and ML classifiers is suitable for the classification of brain tumors. Although the performance of our proposed method is promising, further research needs to be done to reduce the size of the model to deploy on a real-time medical diagnosis system using knowledge distillation approaches.

## Figures and Tables

**Table 1:** Work Related to Brain Tumor Classification.

Author	Type of Solution	Classification Method	Objective	Dataset	Feature Extraction Method	Accuracy
Rajan and Sundar, 2019	Classical Machine Learning-based Solutions	Support vector machine (SVM)	Tumor detection and segmentation	41 magnetic resonance (MR) images	Adaptive Gray-Level Co-Occurrence Matrix (AGLCM)	98%
Kharrat et al., 2010		Hybrid method-Genetic algorithm with SVM	Classification of brain tumor into normal, malignant, and benign tumor	83 MR images	Wavelet-based features	98.14%
Shree and Kumar, 2018		Probabilistic neural network (PNN)	Classification of brain MRI into normal and abnormal	650 MR images	Gray level co-occurrence matrix	95%
Arunachalam and Royappan, 2017		Feed-forward back propagation neural network	Classification of brain MRI into normal and abnormal	230 MR images	Gabor, GLCM, and discrete wavelet transform (DWT)	99.8%
Ullah et al., 2020		Feed-forward neural network	Classification of brain MRI intonormal and abnormal	71 MR images	DWT	95.8%
B. Ural, 2018		PNN	Brain tumor detection	25 MR images	k-mean with fuzzy c-mean (KMFCM)	90%
Preethi and Ashwarya, 2019		Deep neural network (DNN)	Classification of tumor and non-tumor image	20 MR images	GLCM + Wavelet GLCM	99.3%
Francisco et al., 2021	Advanced Deep Learning-based Solutions	Multi-pathway convolutional neural network (CNN)	Brain tumor classification	3064 MR images	CNN	97.3%
Deepak and Ameer, 2019		Deep transfer learning	Classification of glioma, meningioma, and pituitary tumors	3064 MR images	GoogleNet	98%
Ahmet and Mohammad, 2020		CNN models	Brain tumor detection and classification	253 MR images	CNN	97.2%
Das et al., 2019		CNN	Brain tumor classification	3064 MR images	CNN	94.39%
Saed et al., 2017		CNN	Classification of brain MRI into normal and abnormal	587 MR images	CNN	91.16%
Saxena et al., 2019		CNN networks with transfer learning	Binary classification of brain tumor into normal and abnormal	253 MR images	CNN	95%
Paul et al., 2017		Fully connected and CNN	Brain tumor classification	3064 MR images	CNN	91.43%
Hemanth et al., 2019		CNN	MR brain image classification into normal and abnormal	220 MR images	CNN	94.5%

**Table 2:** Kernel types and their required parameters.

Kernel	Equation	Parameters
Linear	$K\left(x_{n},x_{i}\right)=\left(x_{n},x_{i}\right)$	-
Sigmoid	$K\left(x_{n},x_{i}\right)=tanh\left(\gamma \left(x_{n},x_{i}\right)+C\right)$	γ,C
RBF	$K\left(x_{n},x_{i}\right)=exp\left(-\gamma {∥x_{n}-x_{i}∥}^{2}+C\right)$	γ,C

**Table 3:** Details of the dataset.

Types	Number of Class	Training Set	Test Set
BT-small-2c	2	202	51
BT-large-2c	2	2400	600
BT-large-4c	4	2611	653

**Table 4:** Accuracies of pre-trained CNN models with ML classifiers on BT-small-2c dataset (⋆ : top-3 features based on average accuracy).

Deep Feature from the Pre-Trained CNN Model	ML Classifier—Accuracy									
	FC	Gaussian NB	AdaBoost	k-NN	RF	SVM (Linear)	SVM (Sigmoid)	SVM (RBF)	ELM	Average
ResNet-50 feature	0.9216	0.8431	0.8431	0.8627	0.8824	0.8235	0.8824	0.9020	0.9020	0.8736
ResNet-101 feature	0.9216	0.8824	0.8431	0.8235	0.9020	0.8235	0.8824	0.9020	0.8824	0.8736
DenseNet-121 feature	0.9216	0.7647	0.8235	0.9216	0.8824	0.8431	0.8824	0.8627	0.9020	0.8671
DenseNet-169 feature ⋆	0.9608	0.8039	0.8627	0.9020	0.9412	0.9608	0.9608	0.9804	0.9412	**0.9237**
VGG-16 feature	0.8431	0.7451	0.7451	0.7059	0.8431	0.8627	0.8627	0.8039	0.8039	0.8017
VGG-19 feature	0.8235	0.6863	0.7843	0.6863	0.8235	0.8235	0.8235	0.8235	0.9020	0.7974
AlexNet feature	0.9216	0.7255	0.8431	0.7843	0.9020	0.8235	0.8627	0.9020	0.9020	0.8519
Inception V3 feature ⋆	0.9216	0.8824	0.9020	0.8235	0.9412	0.9020	0.9020	0.9020	0.9020	0.8976
ResNeXt-50 feature ⋆	0.9412	0.9020	0.9020	0.9020	0.9216	0.9216	0.9216	0.9216	0.9216	0.9172
ResNeXt-101 feature	0.9216	0.8039	0.8235	0.8235	0.9020	0.8627	0.9020	0.9216	0.9216	0.8758
ShuffleNet V2 feature	0.8431	0.7647	0.9216	0.8627	0.9020	0.9412	0.9412	0.9412	0.9412	0.8954
MobileNet V2 feature	0.8824	0.8431	0.7843	0.8431	0.8824	0.8627	0.8824	0.8824	0.8627	0.8584
MnasNet feature	0.9216	0.7843	0.8235	0.8235	0.9216	0.8431	0.8627	0.8627	0.9020	0.8606
Average	**0.9035**	0.8024	0.8386	0.8281	0.8959	0.8688	0.8899	0.8929	0.8989	

The bold text represents the highest average accuracy of all ML classifier or all deep features.

**Table 5:** Accuracies of pre-trained CNN models with ML classifiers on BT-large-2c dataset (⋆ : top-3 features based on average accuracy).

Deep Feature from the Pre-Trained CNN Model	ML Classifier—Accuracy									
	FC	Gaussian NB	AdaBoost	k-NN	RF	SVM (Linear)	SVM (Sigmoid)	SVM (RBF)	ELM	Average
ResNet-50 feature	0.9767	0.8117	0.9600	0.9767	0.9400	0.9750	0.9750	0.9817	0.9667	0.9515
ResNet-101 feature	0.9767	0.8250	0.9433	0.9733	0.9567	0.9750	0.9733	0.9800	0.9717	0.9528
DenseNet-121 feature ⋆	0.9750	0.8383	0.9600	0.9817	0.9683	0.9683	0.9683	0.9833	0.9817	0.9583
DenseNet-169 feature	0.9750	0.8400	0.9650	0.9783	0.9633	0.9667	0.9650	0.9800	0.9800	0.9570
VGG-16 feature	0.9550	0.7383	0.8833	0.9617	0.9283	0.9517	0.9500	0.9650	0.9550	0.9209
VGG-19 feature	0.9550	0.7067	0.8850	0.9600	0.9300	0.9550	0.9550	0.9633	0.9450	0.9172
AlexNet feature	0.9633	0.7067	0.9200	0.9550	0.9500	0.9400	0.9500	0.9750	0.9633	0.9248
Inception V3 feature	0.9817	0.8317	0.9567	0.9800	0.9567	0.9750	0.9733	0.9883	0.9800	0.9581
ResNeXt-50 feature	0.9717	0.8600	0.9550	0.9817	0.9550	0.9700	0.9683	0.9833	0.9750	0.9578
ResNeXt-101 feature ⋆	0.9783	0.8583	0.9633	0.9833	0.9617	0.9717	0.9717	0.9817	0.9817	**0.9613**
ShuffleNet V2 feature	0.9433	0.8533	0.9533	0.9700	0.9517	0.9617	0.9617	0.9783	0.9700	0.9493
MobileNet V2 feature	0.9667	0.8400	0.9367	0.9700	0.9450	0.9617	0.9617	0.9783	0.9633	0.9470
MnasNet feature ⋆	0.9817	0.8550	0.9467	0.9750	0.9567	0.9700	0.9733	0.9817	0.9833	0.9581
Average	0.9692	0.8127	0.9406	0.9728	0.9510	0.9647	0.9651	**0.9785**	0.9705	

The bold text represents the highest average accuracy of all ML classifier or all deep features.

**Table 6:** Accuracies of pre-trained CNN models with ML classifiers on BT-large-4c dataset (⋆ : top-3 features based on average accuracy).

Deep Feature from the Pre-Trained CNN Model	ML Classifier—Accuracy									
	FC	Gaussian NB	AdaBoost	k-NN	RF	SVM (Linear)	SVM (Sigmoid)	SVM (RBF)	ELM	Average
ResNet-50 feature	0.8760	0.6937	0.6570	0.8576	0.8530	0.8744	0.8760	0.8989	0.8591	0.8273
ResNet-101 feature	0.8867	0.7228	0.6799	0.8438	0.8499	0.8897	0.8897	0.9081	0.8683	0.8377
DenseNet-121 feature	0.8913	0.7106	0.7198	0.8943	0.8744	0.8698	0.8729	0.9158	0.8760	0.8472
DenseNet-169 feature ⋆	0.8959	0.7228	0.7335	0.8821	0.8652	0.8652	0.8729	0.9204	0.8806	0.8487
VGG-16 feature	0.8760	0.6677	0.7106	0.8331	0.8300	0.8606	0.8606	0.8744	0.8423	0.8173
VGG-19 feature	0.8683	0.5942	0.6309	0.8346	0.8377	0.8606	0.8606	0.8790	0.8453	0.8013
AlexNet feature	0.8637	0.6340	0.6554	0.8714	0.8453	0.8652	0.8683	0.9066	0.8361	0.8162
Inception V3 feature	0.8652	0.6708	0.6830	0.8300	0.8132	0.8591	0.8591	0.8867	0.8438	0.8123
ResNeXt-50 feature	0.8744	0.7152	0.6891	0.8775	0.8346	0.8560	0.8576	0.8959	0.8560	0.8285
ResNeXt-101 feature	0.8851	0.6692	0.7198	0.8714	0.8346	0.8744	0.8744	0.8989	0.8744	0.8336
ShuffleNet V2 feature ⋆	0.8637	0.7152	0.7381	0.8637	0.8576	0.8989	0.8989	0.9112	0.8606	0.8453
MobileNet V2 feature	0.8928	0.6983	0.7136	0.8897	0.8423	0.8851	0.8851	0.9158	0.8729	0.8440
MnasNet feature ⋆	0.8851	0.6922	0.7458	0.8928	0.8515	0.8959	0.8959	0.9127	0.8775	**0.8499**
Average	0.8788	0.6851	0.6982	0.8648	0.8453	0.8735	0.8748	**0.9019**	0.8610	

The bold text represents the highest average accuracy of all ML classifier or all deep features.

**Table 7:** Accuracies of ensemble of pre-trained CNN models with ML classifiers on BT-small-2c dataset.

Deep Feature from the Pre-Trained CNN Model	ML Classifier—Accuracy								
	FC	Gaussian NB	AdaBoost	k-NN	RF	SVM (Linear)	SVM (Sigmoid)	SVM (RBF)	ELM
DenseNet-169 feature	**0.9608**	0.8039	0.8627	0.9020	**0.9412**	**0.9608**	**0.9608**	**0.9804**	**0.9412**
Inception V3 feature	0.9216	0.8824	**0.9020**	0.8235	**0.9412**	0.9020	0.9020	0.9020	0.9020
ResNeXt-50	0.9412	**0.9020**	0.9020	0.9020	0.9216	0.9216	0.9216	0.9216	0.9216
(DenseNet-169 + Inception V3) feature	0.9412	0.8627	0.9020	0.8824	0.9020	0.9412	0.9412	0.9608	**0.9412**
(DenseNet-169 + ResNeXt-50) feature	0.9412	**0.9020**	**0.9216**	0.8627	0.9216	0.9216	0.9412	0.9412	**0.9412**
(Inception V3 + ResNeXt-50) feature	0.9412	**0.9020**	0.8824	**0.9412**	**0.9412**	0.9216	0.9412	0.9412	**0.9412**
(DenseNet-169 + Inception V3 + ResNeXt-50) feature	0.9412	**0.9020**	**0.9216**	0.9020	0.9216	0.9020	0.9412	0.9412	0.9216

The bold text represents the highest accuracy for each ML classifier.

**Table 8:** Accuracies of ensemble of pre-trained CNN models with ML classifiers on BT-large-2c dataset.

Deep Feature from the Pre-Trained CNN Model	ML Classifier—Accuracy								
	FC	Gaussian NB	AdaBoost	k-NN	RF	SVM (Linear)	SVM (Sigmoid)	SVM (RBF)	ELM
DenseNet-121 feature	0.9750	0.8383	0.9600	0.9817	0.9683	0.9683	0.9683	0.9833	0.9817
ResNeXt-101 feature	0.9783	0.8583	0.9633	0.9833	0.9617	0.9717	0.9717	0.9817	0.9817
MnasNet feature	0.9817	0.8550	0.9467	0.9750	0.9567	0.9700	0.9733	0.9817	0.9833
(DenseNet-121 + ResNeXt-101) feature	0.9800	0.8733	0.9700	0.9817	0.9667	0.9783	0.9783	0.9833	0.9850
(DenseNet-121 + MnasNet) feature	0.9817	0.8767	0.9633	**0.9850**	0.9683	0.9700	0.9717	0.9783	0.9767
(ResNeXt-101 + MnasNet) feature	**0.9883**	0.8700	0.9633	**0.9850**	0.9667	**0.9850**	**0.9850**	**0.9850**	0.9850
(DenseNet-121 + ResNeXt-101 + MnasNet) feature	**0.9883**	**0.8800**	**0.9750**	0.9817	**0.9717**	0.9783	0.9800	**0.9850**	**0.9867**

The bold text represents the highest accuracy for each ML classifier.

**Table 9:** Accuracies of ensemble of pre-trained CNN models with ML classifiers on BT-large-4c dataset.

Deep Feature from the Pre-Trained CNN Model	ML Classifier—Accuracy								
	FC	Gaussian NB	AdaBoost	k-NN	RF	SVM (Linear)	SVM (Sigmoid)	SVM (RBF)	ELM
DenseNet-169 feature	0.8959	0.7228	0.7335	0.8821	0.8652	0.8652	0.8729	0.9204	0.8806
Shufflenet feature	0.8637	0.7152	0.7381	0.8637	0.8576	0.8989	0.8989	0.9112	0.8606
MnasNet feature	0.8851	0.6922	0.7458	0.8928	0.8515	0.8959	0.8959	0.9127	0.8775
(DenseNet-169 + Shufflenet) feature	0.8959	**0.7504**	0.7427	0.8821	0.8668	0.8668	0.8714	0.9204	0.8744
(DenseNet-169 + MnasNet) feature	0.9142	0.7259	0.7274	**0.9096**	0.8668	**0.9020**	**0.9096**	**0.9372**	0.8790
(Shufflenet + MnasNet) feature	0.8913	0.7305	0.7397	0.8943	**0.8790**	0.8974	0.8974	0.9127	0.8637
(DenseNet-169 + Shufflenet + MnasNet) feature	**0.9158**	0.7397	**0.7534**	**0.9096**	0.8760	**0.9020**	**0.9096**	**0.9372**	**0.8851**

The bold text represents the highest accuracy for each ML classifier.

**Table 10:** Computational complexity of ensemble of pre-trained CNN models with ML classifiers on BT-large-4c dataset.

Deep Feature from the Pre-Trained CNN Model	ML Classifier—Accuracy								
	FC	Gaussian NB	AdaBoost	k-NN	RF	SVM (Linear)	SVM (Sigmoid)	SVM (RBF)	ELM
(DenseNet-169 + Shufflenet) feature	0.0222	0.0214	0.2709	5.0436	0.0148	1.7390	1.9813	2.2653	0.1831
(DenseNet-169 + MnasNet) feature	0.0225	0.0232	0.3070	5.5191	0.0187	1.9650	2.1004	2.5780	0.2184
(Shufflenet + MnasNet) feature	0.0224	0.0186	0.2403	4.3021	0.0170	1.4580	1.4725	2.2544	0.1784
(DenseNet-169 + Shufflenet + MnasNet) feature	0.0229	0.0312	0.4133	7.4238	0.0247	2.6586	2.8507	3.4730	0.2772

## Data Availability

Data are available in publicly accessible repositories which are described in Section 4.1.

## References

[1] Louis D.N., Perry A., Reifenberger G., Deimling A.V., Figarella-Branger D., Cavenee W.K., Ohgaki H., Wiestler O.D., Kleihues P., Ellison D.W. (2016). The 2016 World Health Organization classification of tumors of the central nervous system: A summary. Acta Neuropathol..

[2] Tandel G.S., Biswas M., Kakde O.G., Tiwari A., Suri H.S., Turk M., Laird J.R., Asare C.K., Ankrah A.A., Khanna N.N. (2019). A review on a deep learning perspective in brain cancer classification. Cancers.

[3] Anaraki A.K., Ayati M., Kazemi F. (2019). Magnetic resonance imaging-based brain tumor grades classification and grading via convolutional neural networks and genetic algorithms. Biocybern. Biomed. Eng..

[4] Liu J., Pan Y., Li M., Chen Z., Tang L., Lu C., Wang J. (2018). Applications of deep learning to MRI images: A survey. Big Data Min. Anal..

[5] Mehrotra R., Ansari M.A., Agrawal R., Anand R.S. (2020). A Transfer Learning approach for AI-based classification of brain tumors. Mach. Learn. Appl..

[6] Pereira S., Pinto A., Alves V., Silva C.A. (2018). Brain tumor segmentation using convolutional neural networks in MRI images. IEEE Trans. Med. Imaging.

[7] Popuri K., Cobzas D., Murtha A., Jägersand M. (2012). 3D variational brain tumor segmentation using Dirichlet priors on a clustered feature set. Int. J. Comput. Assist. Radiol. Surg..

[8] Ullah Z., Farooq M.U., Lee S.H., An D. (2020). A Hybrid Image Enhancement Based Brain MRI Images Classification Technique. Med. Hypotheses.

[9] Selvaraj H., Selvi S.T., Selvathi D., Gewali L. (2007). Brain MRI slices classification using least squares support vector machine. Int. J. Intell. Comput. Med. Sci. Image Process..

[10] John P. (2012). Brain tumor classification using wavelet and texture based neural network. Int. J. Sci. Eng. Res..

[11] Bosch A., Munoz X., Oliver A., Marti J. Modeling and classifying breast tissue density in mammograms. Proceedings of the 2006 IEEE Computer Society Conference on Computer Vision and Pattern Recognition (CVPR’06).

[12] Avni U., Greenspan H., Konen E., Sharon M., Goldberger J. (2010). X-ray categorization and retrieval on the organ and pathology level, using patch-based visual words. IEEE Trans. Med. Imaging.

[13] Yang W., Lu Z., Yu M., Huang M., Feng Q., Chen W. (2012). Content-based retrieval of focal liver lesions using bag-of-visual-words representations of single-and multiphase contrast-enhanced CT images. J. Digit. Imaging.

[14] Cheng J., Yang W., Huang M., Huang W., Jiang J., Zhou Y., Yang R., Zhao J., Feng Y., Feng Q. (2016). Retrieval of brain tumors by adaptive spatial pooling and fisher vector representation. PLoS ONE.

[15] Mohammad H., Axel D., Warde F. (2017). Brain tumor segmentation with deep neural networks. Med. Image Anal..

[16] Prastawa M., Bullitt E., Moon N., Van L., Gerig G. (2003). Automatic brain tumor segmentation by subject specific modification of atlas priors1. Acad. Radiol..

[17] Ateeq T., Majeed M., Nadeem A., Syed M., Maqsood M., Rehman Z., Lee J.W., Muhammad K., Shuihua B., Sung W. (2018). Ensemble-classifiers-assisted detection of cerebral microbleeds in brain MRI. Comput. Electr. Eng..

[18] Kharrat A., Gasmi K., Messaoud M., Ben N.B., Abid M. (2010). A hybrid approach for automatic classification of brain MRI using genetic algorithm and support vector machine. Leonardo J. Sci..

[19] Papageorgiou E., Spyridonos P., Glotsos D., Stylios C., Ravazoula P., Nikiforidis G., Groumpos P. (2008). Brain tumor characterization using the soft computing technique of fuzzy cognitive maps. Appl. Soft Comput..

[20] Shree N.V., Kumar T.N.R. (2018). Identification and classification of brain tumor MRI images with feature extraction using DWT and probabilistic neural network. Brain Inform..

[21] Arunachalam M., Royappan S.S. (2017). An efficient and automatic glioblastoma brain tumor detection using shift-invariant shearlet transform and neural networks. Int. J. Imaging Syst. Technol..

[22] Rajan P.G., Sundar C. (2019). Brain tumor detection and segmentation by intensity adjustment. J. Med. Syst..

[23] Kleesiek J., Urban G., Hubert A., Schwarz D., Maier-Hein K., Bendszus M., Biller A. (2016). Deep MRI brain extraction: A 3D convolutional neural network for skull stripping. NeuroImage.

[24] Paul J.S., Plassard A.J., Landman B.A., Fabbri D. (2017). Deep learning for brain tumor classification. Med. Imaging Biomed. Appl. Mol. Struct. Funct. Imaging.

[25] Abiwinanda N., Hanif M., Hesaputra S.T., Handayani A., Mengko T.R. Brain tumor classification using convolutional neural network. Proceedings of the World Congress on Medical Physics and Biomedical Engineering 2018.

[26] Seetha J., Raja S.S. (2018). Brain tumor classification using convolutional neural networks. Biomed. Pharmacol. J..

[27] Hemanth D.J., Anitha J., Naaji A., Geman O., Popescu D.E. (2018). A modified deep convolutional neural network for abnormal brain image classification. IEEE Access.

[28] Balasooriya N.M., Nawarathna R.D. A sophisticated convolutional neural network model for brain tumor classification. Proceedings of the IEEE International Conference on Industrial and Information Systems (ICIIS).

[29] Deepak S., Ameer P.M. (2019). Brain tumor classification using deep CNN features via transfer learning. Comput. Biol. Med..

[30] Çinar A., Yıldırım M. (2020). Detection of tumors on brain MRI images using the hybrid convolutional neural network architecture. Med. Hypotheses.

[31] Khawaldeh S., Pervaiz U., Rafiq A., Alkhawaldeh R.S. (2018). Noninvasive grading of glioma tumor using magnetic resonance imaging with convolutional neural networks. Appl. Sci..

[32] Saxena P., Maheshwari A., Maheshwari S. (2019). Predictive modeling of brain tumor: A Deep learning approach. arXiv.

[33] Xuesong Y., Yong F. (2018). Feature extraction using convolutional neural networks for multi-atlas based image segmentation. Med. Imaging Image Process..

[34] Wicht B. (2017). Deep Learning Feature Extraction for Image Processing. Ph.D. Thesis.

[35] Francisco J.P., Mario Z.M., Miriam R.A. (2021). A Deep Learning Approach for Brain Tumor Classification and Segmentation Using a Multiscale Convolutional Neural Network. Healthcare.

[36] Raja P.M.S., Antony V.R. (2020). Brain tumor classification using a hybrid deep autoencoder with Bayesian fuzzy clustering-based segmentation approach. Biocybern. Biomed. Eng..

[37] Bhuvaji S., Kadam A., Bhumkar P., Dedge S., Kanchan S. Brain Tumor Classification (MRI) Dataset. https://www.kaggle.com/sartajbhuvaji/brain-tumor-classification-mri.

[38] Preethi S., Aishwarya P. (2019). Combining Wavelet Texture Features and Deep Neural Network for Tumor Detection and Segmentation Over MRI. J. Intell. Syst..

[39] Ural B. (2018). A computer-based brain tumor detection approach with advanced image processing and probabilistic neural network methods. J. Med. Biol. Eng..

[40] Zhang X., Zhou X., Lin M., Sun J. Finding Extreme Points in Contours with OpenCV. https://www.pyimagesearch.com/2016/04/11/finding-extreme-points-in-contours-with-opencv.

[41] Goyal M., Goyal R., Lall B. (2019). Learning Activation Functions: A New Paradigm of Understanding Neural Networks. arXiv.

[42] Albawi S., Mohammed T.A., Al-Zawi S. Understanding of a convolutional neural network. Proceedings of the 2017 International Conference on Engineering and Technology (ICET).

[43] Krizhevsky A., Sutskever I., Hinton G.E. (2012). Imagenet classification with deep convolutional neural networks. Adv. Neural Inf. Process. Syst..

[44] Akçay S., Kundegorski M.E., Devereux M., Breckon T.P. Transfer learning using convolutional neural networks for object classification within x-ray baggage security imagery. Proceedings of the 2016 IEEE International Conference on Image Processing (ICIP).

[45] Baltruschat I.M., Nickisch H., Grass M., Knopp T., Saalbach A. (2019). Comparison of deep learning approaches for multi-label chest X-ray classification. Sci. Rep..

[46] Christodoulidis S., Anthimopoulos M., Ebner L., Christe A., Mougiakakou S. (2016). Multisource transfer learning with convolutional neural networks for lung pattern analysis. IEEE J. Biomed. Health Inform..

[47] Kang J., Gwak J. (2019). Ensemble of instance segmentation models for polyp segmentation in colonoscopy images. IEEE Access.

[48] Tajbakhsh N., Shin J.Y., Gurudu S.R., Hurst R.T., Kendall C.B., Gotway M.B., Liang J. (2016). Convolutional neural networks for medical image analysis: Full training or fine tuning?. IEEE Trans. Med. Imaging.

[49] Pan S.J., Yang Q. (2009). A survey on transfer learning. IEEE Trans. Knowl. Data Eng..

[50] He K., Zhang X., Ren S., Sun J. Deep residual learning for image recognition. Proceedings of the IEEE Conference on Computer Vision and Pattern Recognition.

[51] Huang G., Liu Z., Maaten L.V.D., Weinberger K.Q. Densely connected convolutional networks. Proceedings of the IEEE Conference on Computer Vision and Pattern Recognition.

[52] Simonyan K., Zisserman A. (2014). Very Deep Convolutional Networks for Large-Scale Image Recognition. arXiv.

[53] Krizhevsky A. (2014). One Weird Trick for Parallelizing Convolutional Neural Networks. arXiv.

[54] Szegedy C., Vanhoucke V., Ioffe S., Shlens J., Wojna Z. Rethinking the inception architecture for computer vision. Proceedings of the IEEE Conference on Computer Vision and Pattern Recognition.

[55] Xie S., Girshick R., Dollár P., Tu Z., He K. Aggregated residual transformations for deep neural networks. Proceedings of the IEEE Conference on Computer Vision and Pattern Recognition.

[56] Ma N., Zhang X., Zheng H.T., Sun J. Shufflenet v2: Practical guidelines for efficient cnn architecture design. Proceedings of the European Conference on Computer Vision (ECCV).

[57] Sandler M., Howard A., Zhu M., Zhmoginov A., Chen L.C. Mobilenetv2: Inverted residuals and linear bottlenecks. Proceedings of the IEEE Conference on Computer Vision and Pattern Recognition.

[58] Tan M., Chen B., Pang R., Vasudevan V., Sandler M., Howard A., Le Q.V. Mnasnet: Platform-aware neural architecture search for mobile. Proceedings of the IEEE Conference on Computer Vision and Pattern Recognition.

[59] Pedregosa F., Varoquaux G., Gramfort A., Michel V., Thirion B., Grisel O., Blondel M., Prettenhofer P., Weiss R., Dubourg V. (2011). Scikit-learn: Machine learning in Python. J. Mach. Learn. Res..

[60] Freund Y., Schapire R.E. (1997). A decision-theoretic generalization of on-line learning and an application to boosting. J. Comput. Syst. Sci..

[61] Breiman L. (2001). Random forests. Mach. Learn..

[62] Cortes C., Vapnik V. (1995). Support-vector networks. Mach. Learn..

[63] Huang G.B., Zhu Q.Y., Siew C.K. Extreme learning machine: A new learning scheme of feedforward neural networks. Proceedings of the 2004 IEEE International Joint Conference on Neural Networks.

[64] Kaplan K., Kaya Y., Kuncan M., Ertunç H.M. (2020). Brain tumor classification using modified local binary patterns (LBP) feature extraction methods. Med. Hypotheses.

[65] Kaur G., Oberoi A. (2020). Novel Approach for Brain Tumor Detection based on Naïve Bayes Classification. Data Management, Analytics and Innovation.

[66] Minz A., Mahobiya C. MR image classification using adaboost for brain tumor type. Proceedings of the 2017 IEEE 7th International Advance Computing Conference (IACC).

[67] Anitha R., Siva S., Raja D. (2018). Development of computer-aided approach for brain tumor detection using random forest classifier. Int. J. Imaging Syst. Technol..

[68] Gumaei A., Hassan M.M., Hassan M.R., Alelaiwi A., Fortino G. (2019). A hybrid feature extraction method with regularized extreme learning machine for brain tumor classification. IEEE Access.

[69] Khan H.A., Jue W., Mushtaq M., Mushtaq M.U. (2020). Brain tumor classification in MRI image using convolutional neural network. Math. Biosci. Eng..

[70] Polat Ö., Güngen C. (2021). Classification of brain tumors from MR images using deep transfer learning. J. Supercomput..

[71] Ghosal P., Nandanwar L., Kanchan S., Bhadra A., Chakraborty J., Nandi D. Brain tumor classification using ResNet-101 based squeeze and excitation deep neural network. Proceedings of the 2019 Second International Conference on Advanced Computational and Communication Paradigms (ICACCP).

[72] Zhou Y., Li Z., Zhu H., Chen C., Gao M., Xu K., Xu J. Holistic brain tumor screening and classification based on densenet and recurrent neural network. Proceedings of the International MICCAI Brainlesion Workshop.

[73] Saba T., Mohamed A.S., El-Affendi M., Amin J., Sharif M. (2020). Brain tumor detection using fusion of hand crafted and deep learning features. Cogn. Syst. Res..

[74] Ezhilarasi R., Varalakshmi P. Tumor detection in the brain using faster R-CNN. Proceedings of the 2018 2nd International Conference on I-SMAC (IoT in Social, Mobile, Analytics and Cloud)(I-SMAC).

[75] Soumik M.F.I., Hossain M.A. Brain Tumor Classification With Inception Network Based Deep Learning Model Using Transfer Learning. Proceedings of the 2020 IEEE Region 10 Symposium (TENSYMP).

[76] Lu S.Y., Wang S.H., Zhang Y.D. (2020). A classification method for brain MRI via MobileNet and feedforward network with random weights. Pattern Recognit. Lett..

[77] Chakrabarty N. Brain MRI Images for Brain Tumor Detection Dataset. https://www.kaggle.com/navoneel/brain-mri-images-for-brain-tumor-detection.

[78] Hamada A. Br35H Brain Tumor Detection 2020 Dataset. https://www.kaggle.com/ahmedhamada0/brain-tumor-detection.

[79] Krizhevsky A., Sutskever I., Hinton G.E. (2017). Imagenet classification with deep convolutional neural networks. Commun. ACM.

